# Coronary Physiology: Delivering Precision Medicine?

**DOI:** 10.31083/j.rcm2305158

**Published:** 2022-04-27

**Authors:** Laura Maitre-Ballesteros, Laurent Riou, Stephanie Marliere, Marjorie Canu, Estelle Vautrin, Nicola Piliero, Oliviez Ormezzano, Helene Bouvaist, Alexis Broisat, Catherine Ghezzi, Daniel Fagret, Gérald Vanzetto, Loïc Djaïleb, Gilles Barone-Rochette

**Affiliations:** ^1^Department of Cardiology, University Hospital, 38000 Grenoble Alpes, France; ^2^Univ. Grenoble Alpes, INSERM, CHU Grenoble Alpes, LRB, 38000 Grenoble, France; ^3^French Alliance Clinical Trial, French Clinical Research Infrastructure Network, 75018 Paris, France

**Keywords:** coronary physiology assessment, coronary microcirculation dysfunction, precise medicine

## Abstract

Coronary physiological assessment is now widely used to assess epicardial coronary lesions in cath lab. Based on clinical evidence, fractional flow reserve (FFR) is the gold standard method to select whether epicardial coronary lesions need revascularization. While additional epicardial indexes, such as instantaneous wave-free ratio (iFR), are also used for revascularization decision-making, several indexes are now also available to explore the coronary microcirculation. Therefore, coronary physiological assessment now allows to explore the entire coronary tree and offer the potential of precision medicine for patients affected by coronary artery disease (CAD). This paper will provide review of the epicardial and microvascular indexes available for the assessment of coronary physiology. More specifically, the already demonstrated contributions of these indexes in the management of CAD and the role they could play in precision medicine will be reviewed with special emphasis on chronic coronary syndrome.

## 1. Introduction 

Coronary physiological assessment is usually used to assess epicardial coronary lesions in cath lab. Based on clinical evidence, fractional flow reserve (FFR) is the gold standard method to select whether epicardial coronary lesions need revascularization. While additional epicardial indexes, such as instantaneous wave-free ratio (iFR), are also used for revascularization decision-making, several indexes are now also available to explore the coronary microcirculation. Therefore, coronary physiological assessment now allows to explore the entire coronary tree and offer the potential of precision medicine for patients affected by coronary artery disease (CAD).

This paper will provide a review of the epicardial and microvascular indexes available for the assessment of coronary physiology. More specifically, the already demonstrated contributions of these indexes in the management of CAD and the role they could play in precision medicine will be reviewed with special emphasis on chronic coronary syndromes.

## 2. Definition of CAD and Pathophysiology

The relevance of the invasive coronary angiography (ICA) assessment of CAD severity is limited. Indeed, ICA predicts the hemodynamic significance of 40–70% coronary stenoses in less than 50% of cases [1]. This can be explained by the fact that ICA does not allow the functionality of the entire coronary tree to be explored. The coronary vasculature may be divided into two components [2]. First, the macroscopic compartment assessed by ICA constitutes less than 10% of the coronary vasculature and is formed by the epicardial arteries (>400 μm) that have a conductance function. At this level the resistance to coronary flow is minimal in absence of epicardial stenosis. Second constitutes 90% of coronary vasculature, the microvascular compartment is constituted by pre-arterioles (100 to 400 μm), arterioles (40 to 100 μm), and capillaries (<10 μm). Pre-arterioles and arterioles regulate and distribute blood flow with maximum resistance to coronary flow to respond to the demands of tissue metabolism through capillaries. Arteriolar tone maintains coronary blood flow (CBF) constant over a wide range of coronary perfusion pressures, thereby attenuating ischemia during process of obstructive epicardial atherosclerosis. While ICA is unable to assess coronary microcirculation, the clinical manifestation of coronary disease depends on the involvement-possibly simultaneous-of these two compartments. ICA with coronary physiological indexes therefore have higher clinical relevance since they allow the analysis of the whole coronary tree. Several presentations are possible. Obstructive CAD occurs in patients with epicardial atherosclerotic lesions responsible for ischemia due to an increased oxygen demand not covered by a corresponding increase in CBF. Non-obstructive coronary artery disease (NOCAD) represents an alternative clinical presentation, in which the evolutive risk corresponds to plaque rupture or erosion leading to an acute event. Several pathways are responsible for angina in non-obstructive CAD (ANOCA) or ischemia with nonobstructive CAD (INOCA). First, vasospastic angina (VSA), in which epicardial coronary artery are the site of vasospasm impairing coronary flow [3]. Second, coronary microvascular dysfunction (CMVD), whether due to structural abnormalities or the inability of the coronary microcirculation to vasodilate appropriately including vasospasm microvascular. There are several possibilities of structural microcirculatory abnormalities, which may be caused by inward remodeling of arterioles with a decreased lumen, or by capillary rarefaction or even capillary compression by myocardial hypertrophy and fibrosis [2].

## 3. Methods of Coronary Physiology Assessment

Recently published data indicate that inaccurate diagnosis of CAD leads to inappropriate treatment and is associated with major adverse cardiovascular events (MACE), persistent symptoms with reduced quality of life, repeated hospitalizations and unnecessary diagnostic procedures [4]. The key to a precise diagnosis is to explore the whole coronary tree with coronary physiological indexes, which cannot be performed by non-invasive methods [4]. Positron emission tomography (PET), transthoracic echocardiography, and cardiac magnetic resonance can detect CMVD by measure of coronary flow reserve (CFR). However, these techniques do not allow assessment of the relative participation of epicardial and microvascular diseases in the reduction of myocardial blood flow. Consequently, the etiology of ischemia as due to obstructive CAD or CMVD may not be systematically identified [5].

### 3.1 Epicardial Coronary Artery Assessment 

#### 3.1.1 Fractional Flow Reserve 

FFR represents the ratio of maximal myocardial blood flow in the territory supplied by the coronary stenosis being interrogated to maximal myocardial blood flow in the same territory if the considered coronary artery was normal (no stenosis). Accordingly, FFR is derived from the ratio between mean coronary blood pressure distal to a stenosed segment (Pd) and mean proximal coronary pressure (Pa) during maximum CBF and a state of minimum microvascular resistance. Essentially, FFR is computed as Pd/Pa during hyperemia induced by intravenous infusion of adenosine for 3 minutes (140 μg/kg/min), or by intravenous bolus regadenoson (400 μg) [6], or by an adenosine intracoronary bolus injection (100 μg) in the right or left coronary artery (100 μg and 200 μg, respectively) [7], or by a papaverine intracoronary injection in the left or right coronary artery (12 mg and 8 mg, respectively). The correlations between these methods are excellent, but it can be noted that Regadenoson and intracoronary injections are faster methods with fewer side effects, especially flushing [6].

#### 3.1.2 Non-Hyperemic Pressure Ratios (NHPR)

NHPRs obviate the necessity of adenosine administration. iFr (Philips/Volcano) is the first NHPR that have been proposed [8]. The study of the relationship between coronary pressure and coronary flow leads to the determination of a wave-free period which allowed the measurement of the Pd/Pa ratio during an interval in which microcirculatory resistance is constant but not necessarily minimal [9]. This mimics the constant microcirculatory resistance during the hyperaemic state whereby measured pressure is proportional to flow. Several other NHPRs have been developed with variations in the timepoint at which the Pd/Pa ratio is measured such as the resting full-cycle ratio (RFR) (Abbott), the diastolic hyperemia-free ratio (Boston Scientific), and the diastolic pressure ratio (Opsens Medical). It should be noted that NHPRs can be used only with vendor proprietary software. However, all these indexes appear comparable [10].

#### 3.1.3 Hyperaemic Stenosis Resistance (HSR)

Even though the severity of the epicardial and microcirculatory damage are not related [11], the status of the microcirculation will influence the FFR measurement [12]. The HSR index is calculated using the formula (Pa - Pd)/average peak velocity (APV) during maximal hyperaemia, and is defined as the resistance provided by the assessed coronary lesion [13]. Although HSR uses flow velocity measurement that depends on epicardial vasculature and microcirculation, it is an index only of epicardial lesions. A HSR value <0.80 mmHg/cm/sec is considered to indicate a significant epicardial stenosis [14].

#### 3.1.4 Angiography-Derived FFR 

Advances in computational power have allowed the development of angiography-derived FFR obviating the need for hyperemia and for a pressure wire. Angiography-derived FFR combines two projections to provide a 3D quantitative coronary angiography, which allows the reconstruction of the specific coronary geometry. An analysis using computational fluid dynamic (CFD) techniques or mathematical formulas then provides a rapid estimation of the pressure drop across a lesion. Angiography-derived FFR demonstrated excellent performances for the diagnosis of hemodynamically significant stenoses defined by FFR <0.80 [15]. Several angiography-derived FFR software packages have been developed. Specifically, Quantitative Flow Ratio (QFR), Cardiovascular Angiographic Analysis Systems for vessel Fractional Flow Reserve (CAAS vFFR), and FFRangio system are angiographically derived estimates of FFR with comparable performances [16]. Suboptimal performances of angiography-derived FFR have been shown for lesions located at bifurcations, for ostial lesions, or for lesions of left main coronary artery. Angiography-derived FFR is also dependent on the quality of angiographic images. It is recommended to have a good catheter engagement in order to optimize contrast artery opacification, to have two optimal angiographic projections >${\mathrm{25}}^{\circ }$, to avoid excessive movement of the X-ray tube, and to use a zoom that cuts parts of the coronary to be analyzed. Like NHPR, the main scientific limitation and challenge in these technologies consists in the unassessed variability of the resistance of the coronary microvascular bed which is not being evaluated.

#### 3.1.5 FFR Derived from Computed Tomography (FFR CT)

As for the angiography-Derived FFR several steps will be necessary to obtain the FFT CT from the Coronary computed tomography angiography (CCTA) imaging. First step, with CCTA image data set an anatomic model of coronary arteries is performed. Then a physiologic model of coronary circulation is produced. Resting coronary flow is modelled on the basis of myocardial mass, and maximal hyperemia is modelled in agreement with the expected reduction in resistance if adenosine injection would be achieved. Finally, supercomputers use computational fluid dynamics methods to measure FFR CT. The HeartFlow FFR CT software (${\mathrm{FFR}}_{\text{CT}}$, HeartFlow Inc, Redwood city, CA, USA) is the most successful technology that has demonstrated its diagnostic performance compared to FFR in 3 significant studies [17, 18, 19]. The diagnostic performance is interesting compared to other non-invasive tests. The PACIFIC trial showed it comparable to PET and superior to SPECT [20]. The use in real life has also been evaluated in a large register prospective multicenter registry [21]. The weakness remains an off-line analysis. FFRCT analysis is only available at a central laboratory in California. CCTA image data set must be sent to post processing which still takes 1 to 4 hours. In addition to the cost of process, the performance also depends strongly on the quality of the image. Motion artifact, severe calcification, and stenting decrease analyzability. For example, in PACIFIC trial, analyzability of FFRCT was only 75% at the patient level [20].

### 3.2 Whole Coronary Tree Assessment

#### 3.2.1 Assessment of Endothelial Dysfunction and Spasm

Acetylcholine provides an endothelium-dependent stimulation. In normal individuals, acetylcholine causes the vasodilatation of epicardial and micro vessels. Paradoxical vasoconstriction occurs in patients with endothelial dysfunction or vasospastic angina. Acetylcholine challenges endothelium-dependent microvascular function. Acetylcholine is usually administered by sequential manual infusion at progressive concentrations of 2 μg, 20 μg, 100 μg and 200 μg over a period of 3 minutes via the diagnostic catheter used for the assessment of the left coronary artery (LCA). The assessment of the right coronary artery is performed when the LCA shows no abnormal result and a dose of 50 μg is then applied prior to 300 mg of glyceryl trinitrate. Manual infusion should be slow (1–2 mL/min). It is more straightforward but possibly less standardized than mechanical sequential infusion. Indeed, mechanical infusion pump can infuse (1 mL/min for 2 minutes) precise progressive concentration of 0.182, 1.82, and 18.2 μg/mL (${\mathrm{10}}^{-6}$, ${\mathrm{10}}^{-5}$, and ${\mathrm{10}}^{-4}$ mol/L, respectively). After each dose, an angiogram is performed. Two criteria are used for diagnosing endothelial dysfunction. Following intracoronary injection of any dose of acetylcholine, endothelial-dependent microvascular dysfunction is defined as a change CBF ≤50% and epicardial endothelial dysfunction is defined as a reduced coronary artery diameter ≥20%. The method of CBF and coronary flow reserve (CFR) computation are detailed below. The change in CBF is provided by the equation *(peak at Ach CBF-baseline CBF)/(baseline CBF)*.

Using Doppler data. It is possible to use the bolus thermodilution technique to diagnose endothelial-dependent microvascular dysfunction through the assessment of endothelial coronary flow reserve with acetylcholine (eCFR) <1.5 [22].

Progressive concentrations of acetylcholine may induce epicardial or microcirculatory spasm. VSA and microvascular spasm (MVS) are defined as follows:

VSA: angina symptoms, ischemic ECG modification and ≥90% constriction in epicardial artery,

MVS: angina symptoms, ischemic ECG modification (≥1 mm) and constriction in epicardial <90%.

It should be noted that acetylcholine test patients with microvascular spasm or a history of myocardial infarction with nonobstructive coronary arteries (MINOCA) have a higher risk of myocardial infarction and recurrent chest pain requiring hospitalization at follow-up despite appropriate post-test therapy [23].

#### 3.2.2 CFR by Endothelium-Independent Stimulation 

CFR provides information on both the epicardial and microvascular compartments by quantifying the ratio of hyperemic to resting CBF. Maximal hyperemia is induced by the intravenous infusion of 140 μg/kg/min of adenosine. The assessment of CFR using thermodilution tends to overestimate values than those measured using Doppler [24]. The absolute cut-off values are <2.0 and <2.5 by thermodilution and Doppler, respectively [25]. CFR reflects the vasodilator capacity of the coronary circulation and does not allow to differentiate the epicardial or microcirculatory involvement. CFR has less reproducibility than FFR due to its dependence on systemic haemodynamics (diastolic time, intramyocardial pressure) and to the high variability of basal flow [26].

The vasodilatory capacity of whole coronary tree is therefore measured using two distinct methods, i.e., acetylcholine testing in order to monitor changes in CBF or eCFR, and adenosine testing in order to quantify CFR. The potentially complementary nature of both remains to be determined.

### 3.3 Coronary Microcirculation 

#### 3.3.1 Measure of CBF 

The principles of CBF measurement must be known in order to understand how to obtain the coronary microcirculation indexes. The microcirculation is invisible to all imaging techniques, which explains that functional tests are the only methods of analysis. The determination of CBF can be performed according to two methods.

Based on bolus thermodilution modelling, flow can be calculated from the mean time it takes a fixed vascular volume to travel from an injector to a sensor (Tmn). De Bruyne *et al*. [27] and Pijls *et al*. [28] conducted the bolus thermodilution technique in animal model and human and found a strong correlation between 1/Tmn and absolute CBF. Absolute CBF assessment is possible using the continuous thermodilution method. It requires a dedicated monorail infusion (RayFlow, Hexacath, Paris, France), an infusion pump, a pressure/temperature wire (PressureWire, Abbott Vascular, Santa Clara, California) and a dedicated software (CoroFlow, Cardiovascular System, Coroventis Research, Uppsala, Sweden). Absolute CBF in mL/min is then given by the equation:

1.08 × Ti/T × Qi,

Qi is the infusion rate of saline at room temperature (20 mL/min). T is the temperature of blood (in ^∘^C) mixed with saline in the distal part of the vessel. Ti is the temperature (in ^∘^C) of the saline as it enters the coronary artery; and the constant 1.08 accounts for the densities and specific heat of blood and saline. Saline infusion with RayFLow induces maximal hyperemia within seconds, which obviates the need for adenosine [29]. It has been shown that absolute CBF and resting resistance can be achieved by continuous thermodilution and a fixed rate of 10 mL/min of saline [30]. This technique reduces the variability of calculated CFR [31].

Hyperemic APV measured using Doppler is used as a surrogate of absolute CBF. Because the Doppler signal provides only flow velocity, quantification of volumetric flow requires exact knowledge of the vessel lumen size, which can be obtained by quantitative coronary angiography (QCA) or intravascular ultrasound (IVUS). The simultaneous measurement of the vessel cross-sectional area and mean velocity (Vmean) allows to CBF in mL/min. Doppler-based wireline systems measure APV. To calculate the mean Vm, a constant coefficient of 0.5 is used, i.e., mean Vm = 0.5 × APV. However, this coefficient is not valuable in the case of pulsatile flow, which is why this method only gives a surrogate for the absolute CBF.

#### 3.3.2 Index of Microcirculatory Resistance (IMR) 

The index of microcirculatory resistance (IMR) is based also on the bolus thermodilution principle. IMR is calculated by: *Pd × Tmn*. IMR is measured with a pressure/temperature wire (PressureWire, Abbott Vascular, Santa Clara, California). In case of significant epicardial stenosis, the IMR value is corrected using Yong’s formula *(Corrected IMR = Pa × Tmn × ([1.35] × Pd/Pa – 0.32) * [32]. A clear cut-off for the diagnosis of CMVD is IMR ≥25 [33]. The variability of IMR quantification represents a limit. It is due to the fact that the measurement depends upon the operator’s bolus injection technique. However, IMR does not depend upon resting measurements or on myocardial mass.

#### 3.3.3 Hyperaemic Microvascular Resistance (HMR)

HMR is measured using a Doppler-equipped guidewire (ComboWire XT; Philips Volcano, San. Diego, CA, USA). It is determined by: *Pd/Hyperemic APV.* There is no clear cut-off for the diagnosis of CMVD. HMR >1.9 or ≥2.5 mmHg/cm/s have been proposed [34, 35].

#### 3.3.4 Resistive Reserve Ratio (RRR)

RRR represents the vasodilatory capacity of microvasculature during hyperemia. It is calculated using the following validated equation [36]: *RRR = BRI/IMR*. The baseline resistance index (BRI) is a measure of the coronary microcirculatory resting tone and is calculated using the formula: *Pd Baseline × Tmn Baseline * [37]. It can be performed with either the bolus or doppler thermodilution techniques. There is no clear cut-off for the diagnosis of CMVD but RRR <2.62 by Doppler was associated with a 1.6-fold higher risk of death in patients with angina or ischemia with NOCAD [38].

#### 3.3.5 Instantaneous Hyperemic Diastolic Velocity Pressure Slope (*IHDVPS*)

The simultaneous acquisition of phasic pressure and flow velocity signals is required to measure IHDVPS. However, this approach is rather complicated in terms of instrumentation. IHDVPS correlates significantly with arteriolar obliteration, capillary density, or arteriolar density [39]. IHDVPS is defined as the slope (β-coefficient) of the relationship between hyperaemic intracoronary pressure and flow in mid-to end diastole, which is displayed by a single regression line (y = a + βx) expressed in cm/s/mmHg. IHDVPS provides a combined view of the arteriolar and capillary domains. IHDVPS normal range has not been established.

#### 3.3.6 Zero-Flow Pressure (Pzf) 

Pzf is extrapolated from the regression line of the relationship between hyperaemic intracoronary pressure and flow in mid- to end diastole and is defined as the intercept of the regression line with the pressure axis. Pzf represents the distal coronary pressure in the theoretical situation of coronary flow cessation. Pzf measured after percutaneous coronary intervention (PCI) is a better predictor of the extent of myocardial infarction than HMR or IMR [40] but no normal range has been established. In addition, the methodology required for Pzf assessment is complex with important offline postprocessing.

#### 3.3.7 Wave Intensity Analysis (WIA)

A wave is a change in pressure and flow that propagates along a blood vessel. There are four wave types: forward compression waves (FCW), forward decompression wave (FDW), backward compression wave (BCW), and backward decompression wave (BDW). The units of wave intensity (Watts/$m^{2}$ or J/sec/$m^{2}$) reflect the rate at which the wave energy passes through a given cross-section of a coronary vessel. BDW originates from the microcirculation and is supposed to quantitatively reflect the re-expansion of the intramyocardial network in early diastole, thereby reflecting in turn capillary density [41]. Here gain a normal range has not been established yet.

The main common problem with the above-described techniques (HMR, IHDVPS, Pzf, WIA) using the Doppler method is that an optimal Doppler signal is obtained in only 69% of the patients [42]. Repositioning and the use of an intracoronary microcatheter to stabilize the position can improve signal quality.

#### 3.3.8 Absolute Resistance, and Microvascular Resistance Reserve (MRR)

Once again, Ohm’s law with ratio of pressure and flow provides absolute resistance expressed in Wood units. The following parameters may then be computed:

*total coronary resistance = Pa/absolute CBF*

*epicardial resistance = Pa-Pd/absolute CBF*

*microvascular resistance = Pd/absolute CBF*

Absolute CBF and resistance as assessed using continuous thermodilution display high reproducibility and low intraobserver variability [43]. A strong agreement has been observed with [^15^O] $H_{2}$O PET–derived flow and resistance [44] following normalization for the myocardial mass of the perfused territory with different algorithms applied to cardiac CT data [45, 46] or intracoronary physiological data [47]. However, the range of normal absolute resistance values have not been extensively investigated yet. Due to interindividual variability that persisted even after normalization for myocardial mass of the perfused territory, the measures are therefore less well suited for individual clinical decision.

Microvascular resistance reserve (MRR) is a novel index which presents several advantages. Because it uses the continued thermodilution method and since it relies on baseline and hyperemic measurements in the same epicardial territory, MRR is independent of myocardial mass, and operator independent. MRR is obtained as follows:

*MRR = Absolute resistance at rest/absolute resistance at hyperemia*

*Absolute resistance at rest = Pa rest/absolute CBF rest*

*Absolute resistance at hyperemia = Pd Hyperemia/absolute CBF hyperemia*

*MRR = (absolute CBF hyperemia/absolute CBF rest) × (Pa rest/Pd hyperemia)*

*MRR = CFR × (Pa rest/Pd hyperemia)*

If MRR is expressed in terms of CFR and FFR the equation is:

*MRR = CFR/FFR × (Pa rest/Pa hyperemia).*

By continue thermodilution method: Pa rest = Pa hyper and final equation is:

*MRR = CFR/FFR*

MRR represents a very promising index since it is specific for the microcirculation, and independent of autoregulation and epicardial resistance [31].

#### 3.3.9 Angiography-Derived IMR 

An elegant use of the QFR in hyperemia has allowed to obtain an angiography-derived IMR [48], which is obtained as follows:

*IMR = Pd hyperaemia × Tmean hyperaemia*

*IMR = Pa hyperaemia × Pd hyperaemia/Pa hyperaemia × Tmean hyperaemia*

*Pd hyperemia/Pa hyperemia ~ QFR*

Tmean hyperemia can be expressed as the ratio between the number of frames (Nframes) that the contrast agent travels from the guiding catheter to a distal marker of the pressure wire to the acquisition rate (fps).

*Angiography-Derived IMR = Pa hyperemia × QFR × (Nframes hyperemia/fps)*

Data post-processing allows to obtain an angiography-derived IMR value without the need for hyperemia. The initial steps are similar to those used for QFR, a 3D reconstruction of the coronary artery and estimation of QFR was performed using CFD. The estimated hyperemic Pa is calculated according to the mean arterial pressure (MAP) with the following weighting:

*MAP × 0.2 when MAP >95 mm Hg and MAP × 0.15 when MAP <95 mm Hg.*

Finally, angiography-derived IMR is computed using the equation:

*Angiography-Derived IMR = Pa hyperemia × QFR × (Nframes hyperemia/fps)*.

Vdiastole is the resting flow velocity during diastole and is derived of the TIMI frame count method multiplied by K which is the constant to adjust the difference between resting and hyperemic flow velocity. Vessel length is determined by the length of vessel opacified by the contrast from the ostium to the distal part [49]. These data are currently monocentric and additional data are needed.

### 3.4 General Comparison between Methods 

Several techniques are needed to explore the whole coronary tree. The analysis of endothelial function and the detection of spasm by the acetylcholine test must be used more and more systematically. For the other indexes, each index explores a part of the coronary tree. At this time, it is not possible to say which technique is the best. However, the interventional cardiologist must know the strengths and weaknesses of each technique and choose and implement a strategy exploring the whole coronary tree through coronary physiology for all his patients. So, each method has strengths and weaknesses that are summarized in Table 1.

**Table 1.** **Strengths and weaknesses of coronary physiology assessment**.

	Advantages	Limitations
FFR	- Best evidences	- Guidewire: cost, complication
	- Prognostic studies available	- Hyperemia: cost and side effect of adenosine
		- Increase time of the procedure
iFR	- Validated by non-inferiority studies vs. FFR	- Guidewire: cost, complication
	- Hyperemia independent	- Increase time of the procedure
	- Quicker than FFR	- Specific software required
Other NHPR	- Hyperemia independent	- Guidewire: cost, complication
	- Quicker than FFR	- Increase time of the procedure
		- Specific software required
		- No evidence regarding outcome prediction
HSR	- Stenosis resistance based on a combination of intracoronary pressure and flow velocity	- Guidewire: cost, complication
		- Increase time of the procedure
		- Specific software required
QFR, CAAS	- Hyperemia independent	- No evidence for outcomes
vFFR, FFRangio system	- No pressure wire	- Specific software required
	- Quicker than FFR if high expertise in post treatment software	- Precise acquisition of angiography
		- Manual correction by expert
FFR CT	- Non invasive	- Cost
	- Increase performance of CCTA	- Off line analysis
CFR	- Study all coronary tree	- Overall assessment (macro and microcirculation)
	- Prognostic performance	- Variability: intrinsic + variable resting condition
		- Guidewire: cost, complication
		- Hyperemia: cost and side effect of adenosine
		- Increase time of the procedure
IMR	- Microcirculation study	- Guidewire: cost, complication
		- Hyperemia: cost and side effect of adenosine
		- Increase time of the procedure
HMR	- Microcirculation study	- Guidewire: cost, complication
		- Hyperemia: cost and side effect of adenosine
		- Increase time of the procedure
		- Doppler: additional cost, Doppler signal not analyzable (30% of patients)
		- No cutoff value
RRR	- Microcirculation study	- Guidewire: cost, complication
		- Hyperemia: cost and side effect of adenosine
		- Increase time of the procedure
		- No evidence regarding outcome prediction
		- No cutoff values
		- Long-lasting procedure
Absolute CBF and resistance	- Operator-independent	- Dependent upon myocardial mass
		- Additional cost
		- Increase time of the procedure
MRR	- Operator-independent	- Additional cost
	- Independent from autoregulation and myocardial mass	- Increase time of the procedure
IHDVPS	- More targeted (theoretically) of Microcirculation study	- Doppler: additional cost, Doppler signal not analyzable (30 % of patients)
Pzf		- Specific equipment required
WIA		

CAAS vFFR, Cardiovascular Angiographic Analysis Systems for vessel Fractional Flow Reserve; CBF, Coronary blood flow; CFR, Coronary flow reserve; CT, computed tomography; FFR, fractional flow reserve; HMR, Hyperaemic Microvascular Resistance; HSR, Hyperaemic Stenosis Resistance; iFR, instantaneous wave-free ratio; IHDVPS, Instantaneous Hyperemic Diastolic Velocity Pressure Slope; IMR, Index of Microcirculatory Resistance; MRR, Microvascular Resistance Reserve; Pzf, Zero-Flow Pressure; QFR, Quantitative Flow Ratio; RRR, Resistive reserve ratio; WIA, Wave Intensity Analysis.

## 4. Demonstrated Contribution of Coronary Physiological Indexes to the Management of CAD Through Randomized Trials

### 4.1 For Epicardial Stenotic Lesions 

#### 4.1.1 FFR and IFR 

Coronary physiological assessment now plays a major role in the decision for PCI revascularization. According to the recommendations, a physiological assessment must be performed before revascularization, when the location of ischemia is not documented for 50% and 90% stenosis by visual estimation or in patients with multivessel CAD [50]. FFR and IFR have been shown to be useful in large randomized studies and should be used as a priority. Accordingly, FFR is currently considered the gold standard because its use has been validated in several large randomized studies. The results of the FAME-1 [1] and FAME-2 trials [51, 52] have demonstrated a clinical benefit in using FFR with a cut off ≤0.8 to guide PCI revascularization. Interestingly, the first major clinical trial using FFR, the DEFER trial, have showed that an FFR-guided PCI strategy is effective and safe in patients >15 years-old [53]. A meta-analysis showed the benefit of FFR-guided PCI over medical therapy alone on the combined end point of cardiovascular death and myocardial infarction [54].

The two largest randomized trials showed that iFR-guided PCI was noninferior to FFR-guided PCI in rates of MACE at 12 months [55, 56]. These results prompted the appearance of iFR in the recommendations using a cut off ≤0.89 [50]. It is regrettable that iFR has less evidence than FFR on long-term results.

Discordance between FFR and iFR appears in an average of 20% of cases. This discordance results from interactions between clinical characteristics, severity or shape of the stenosis [57, 58], variability in coronary physiological responses to rest and hyperemia [59], and location of the stenosis [60]. Indeed, for the localization some studies have shown that lesion of left main (LM) might be associated with a higher discordance between iFR and FFR values (iFR-/FFR+) questioning the use of iFR in this setting. The DEFINE-LM registry shows that deferral or perform revascularization of LM stenosis based on iFR appears to be secure [61].

Finally, because the discordance between FFR and iFR does not lead to differences in outcomes [59], it is more interesting to discuss of practical use on specific clinical setting. For example, iFR could be an attractive alternative to FFR in patients with multivessel CAD to perform multiple measurements without inducing hyperemia. There may also be a reluctance by many operators to use vasodilators in patients with bradycardia or hypotension. iFR could be an alternative. The value of iFR in cases of abnormal coronary microcirculation is suggested especially in acute coronary syndrome (ACS) and in patients with severe aortic stenosis. However, the evaluation of non-infarct-related arteries in the early phase of ACS creates diagnostic problems for the FFR but also for the IFR. The first is explained to a blunted hyperemia associated to ACS and the second by increased coronary resting flow on territory of remote myocardial infarction with compensatory hyperkinesia [62]. Recently, FLOWER MI trial failed to prove that a complete revascularization that is guided by FFR is superior to an angiography-guided procedure in STEMI patients [63]. If we can evoke the problem of the use of the FFR at the time of primary PCI due to blunted hyperemia associated to ACS [62], these results were more explained to a lack of statistical power due to lower-than-expected incidence of events. Probably future studies will precise the optimal time to use FFR or iFR to evaluate non-infarct-related arteries. In the setting of patient with severe aortic stenosis, conflicting data of evolution of FFR after TAVR implantation create debate with either a decrease in FFR after TAVR implantation [64] or stability [65]. So, further studies are needed in this area to clarified use of iFR and FFR.

To complete, several recent studies on the FFR appear with negative results in patients with multivessel CAD. However, negative results are probably due to reasons other than a questioning of the FFR performance.

The FUTURE trial compared an FFR-guided strategy with a traditional non-FFR strategy in the treatment of multivessel CAD. The trial was stopped prematurely by data safety and monitoring board due to higher all-cause mortality associated with FFR-guided strategy. This observation was not confirmed by the intention-to treat analysis at 1-year follow-up. At follow up, there was no significant difference between both strategies [66]. It is really difficult to conclude given the limited statistical power of the study. The higher all-cause mortality initial was probably due to chance.

The results of the FAME 3 trial are more instructive [67]. FAME 3 was a multicenter, international, noninferiority trial, patients with multivessel CAD were randomly assigned to undergo CABG or FFR-guided PCI with zotarolimus-eluting stents. The composite primary end point was death from any cause, myocardial infarction, stroke, or repeat revascularization at 1 year. FFR-guided PCI was not found to be noninferior to CABG. Probably it is not the performance of FFR that can be questioned. FAME 3 confirms that CABG is the best treatment for multivessel CAD [50]. Indeed, previous randomized clinical trials that assessed use of FFR were performed in patients eligible for PCI. Patients with multivessel CAD presented often long and severe diffuse lesions and PCI tends to be more appropriate for focal disease where the FFR is known to be more efficient [57, 58].

#### 4.1.2 QFR

QFR has an advantage over alternative angiography-derived FFR indexes since the publication of the FAVOR III China study results. This prospective study included 3825 patients from China in which the QFR-guided PCI was used with a cut off ≤0.89 and compared with an angiography-guided PCI. All cause death, MI and ischemia driven revascularization were the composite endpoint and occurred in 5.8% (11/1913) of patients in the QFR group compared to 8.8% (167/1912) in the angiography group (HR 0.65, 95% CI 0.51–0.83, *p* = 0.004) at 1 year [68]. The results of FAVOR III EJ (NCT03729739) will be more interesting since the study design investigates whether QFR-guided PCI will be non-inferior at 12 months compared to an FFR-guided PCI.

#### 4.1.3 FFR CT 

The number of randomized studies is still limited. The PLATFORM trial, which evaluated FFR CT in patients with planned ICA for chronic coronary syndrome. FFR CT was a feasible and safe with a significantly lower rate of NOCAD at ICA. At 1-year follow-up, the FFR CT strategy appeared lower cost than the ICA strategy with an equivalent cardiac event rate and the same level of quality of life [69, 70]. The SYNTAX III trial, in patients with left main or 3-vessel coronary artery disease, showed that CCTA analysis made the same revascularization decision as ICA analysis, and that the use of FFR CT changed the decision in 7% of cases [71]. In FORECAST trial, use of FFR CT reduced ICA, and did not differ significantly from control group in cost or clinical outcomes [72]. However, control group had mainly CCTA (63%) as the initial test. Studies with adequate statistical power to compare the performance of FFR CT with other non-invasive tests in the management of chronic coronary syndrome are expected.

### 4.2 For Coronary Microcirculation 

Up to 70% of patients with angina or myocardial ischemia will have a NOCAD at ICA [73]. The underlying cause of ANOCA (or INOCA) should be assessed systematically using invasive coronary physiology [25, 74]. The CorMicA randomized trial showed that the use of coronary physiological measures for the assessment of microvascular and/or vasospastic angina to introduce stratified medicine in patients with stable angina and NOCAD is superior to standard care. Coronary physiological assessment was found to be relevant to introduce a tailored treatment that improved symptoms, quality of life, and decreased unnecessary ICA [75, 76]. The use of coronary physiological measurements in INOCA, also called interventional diagnostic procedures, follows an expert consensus developing diagnostic and therapeutic strategies [77]. This expert consensus allows to choose the sequence of testing. Performing adenosine testing first without nitroglycerin is the most suitable choice due to the pharmacodynamics of vasoactive drugs [78]. The sequence consists in coronary angiography and FFR in order to exclude obstructive CAD, then the assessment of vasodilatation is performed first by adenosine and then followed by acetylcholine test. Then, patient can be classified according to endotypes. Endotype 1 is the microvascular angina (MVA) (abnormal vasodilatation and/or microvascular spasm (MVS)); Endotype 2 is VSA (epicardial spasm); endotype 3 is a mixed MVA and VSA (epicardial spasm + abnormal vasodilatation); and endotype 4, extra-cardiac chest pain. This classification follows different therapeutic recommendations.

The diagnostic criteria are:

• VSA: angina symptoms, ischemic ECG modification and ≥90% constriction in epicardial artery.

• MVA: angina, no obstructive CAD plus objective evidence of coronary microvascular dysfunction (MVS and/or CFR <2 and/or IMR ≥25).

• MVS: angina, ischemic ECG modification (≥1 mm) and absence of constriction in epicardial artery <90%.

• Mixed MVA and VSA: angina with no obstructive CAD plus both evidence of invasive coronary microvascular dysfunction and epicardial vasospasm to acetylcholine (≥90% epicardial constriction).

• Extracardiac chest pain: normal results of coronary physiology assessment.

• Endothelial dysfunction is defined by ≥20% luminal constriction during acetylcholine test.

## 5. Towards Precision Medicine

**Fig. 1.**
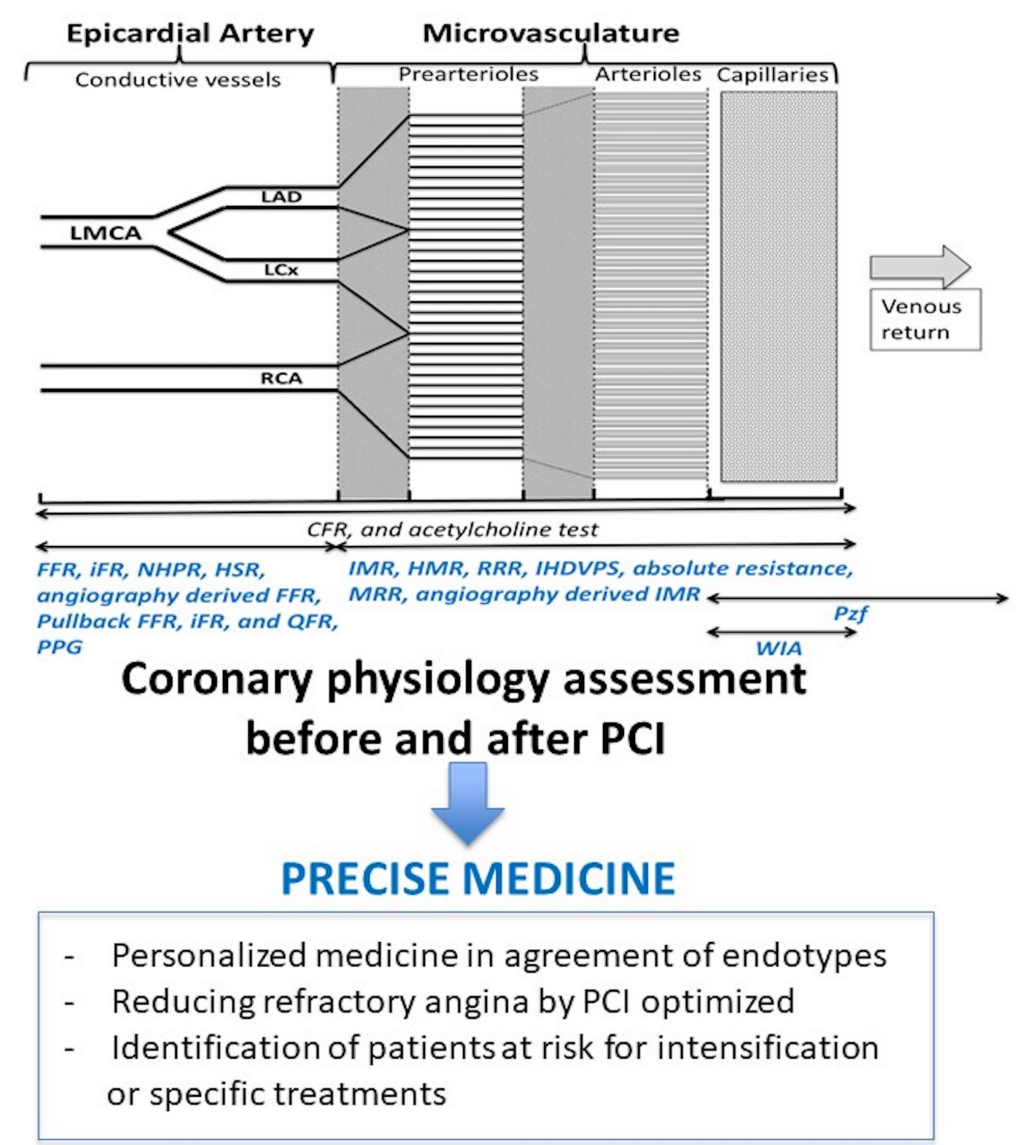
**Proposition of utilization of coronary physiology assessment to precise medicine**. CFR, Coronary flow reserve; FFR, fractional flow reserve; HMR, Hyperaemic Microvascular Resistance; HSR, Hyperaemic Stenosis Resistance; iFR, instantaneous wave-free ratio; IHDVPS, Instantaneous Hyperemic Diastolic Velocity Pressure Slope; IMR, Index of Microcirculatory Resistance; MRR, Microvascular Resistance Reserve; PPG, Pullback pressure gradient; Pzf, Zero-Flow Pressure; QFR, Quantitative Flow Ratio; RRR, Resistive reserve ratio; WIA, Wave Intensity Analysis.

The term “precision medicine” refers to a medical concept where diseases are managed according to the individual characteristics of each patient. Precise medicine is applicable for prevention, diagnostic and therapeutic strategies. Oncology is far ahead of cardiology in this field by using critical data sources ranging from genomics, transcriptomics, proteomics, and metabolomics. In cardiology, the use of coronary physiological assessment already allows the implementation of personalized medicine in several situations (Fig. 1).

### 5.1 Personalized Medicine in Agreement of Endotypes

CorMicA trial has paved the way for precision medicine in patients with ANOCA or INOCA by identification of endotypes. For the CMVD, an even more specific distinction can be recognized, i.e., structural and functional CMVD. Functional CMVD is distinguished by elevated CBF at rest, due to increased nitric oxide synthase (NOS) activity, and by normal maximal CBF during exercise. Alternatively, patients with structural CMVD present an endothelial dysfunction, which results in decreased peak CBF during exercise and normal CBF at rest [79, 80]. Whether functional and structural CMVD may translate into distinct prognosis or require distinct treatments warrants further investigation.

### 5.2 Reducing Refractory Angina by PCI Optimized 

Following PCI and despite adequate anti-ischemic therapy, 20% to 30% of patients continue to present angina [81]. Assessing coronary physiology after angioplasty will provide access to data useful for the improvement of patient management. First, coronary physiological assessment can detect microvascular and/or vasospastic angina which may be associated with epicardial stenosis and which will require an adjusted treatment [82]. Second, physiological indexes can be used to detect and understand mechanisms of suboptimal PCI results associated with wrong prognosis. Thus, an FFR <0.86, an iFR <0.89, or a QFR <0.89 post-angioplasty may be considered pejorative [83]. There are several reasons for incorrect FFR values after PCI such as stent-related cause (stent edge dissection or underexpansion), significant stenosis located proximally to the target PCI, diffuse vessel disease, or coronary spasm pseudo-stenoses caused by the pressure guidewire [83]. In 24% of cases with iFR <0.89 a suboptimal result after PCI is explained mainly by focal lesions outside the stent [84]. Once the alert is given, these causes must be identified and managed using a dedicated treatment. Coronary physiological assessment can help with FFR pullback, IFR scout pullback or QFR virtual pullback to understand the problem by determining if it is focal and whether it requires an additional stent. In such cases, endocoronary imaging such as IVUS or OCT may be used. In case of diffuse the damage, an intensification of cardioprotective treatments will be required, especially anti-ischemic treatments, together with the prevention of the possibility of refractory angina, in which case education and cardiovascular rehabilitation can be very useful. In this setting, the pullback pressure gradient (PPG) index is interesting because it is a continuous measure with values close to 0 indicating diffuse CAD, whereas those close to 1 suggest focal CAD. PPG index is given by the equation:

{*maxPPG 20 mm/Δ FFR vessel + (1-Length with functional disease (mm)/Total vessel length (mm))*}*/2*

**Fig. 2.**
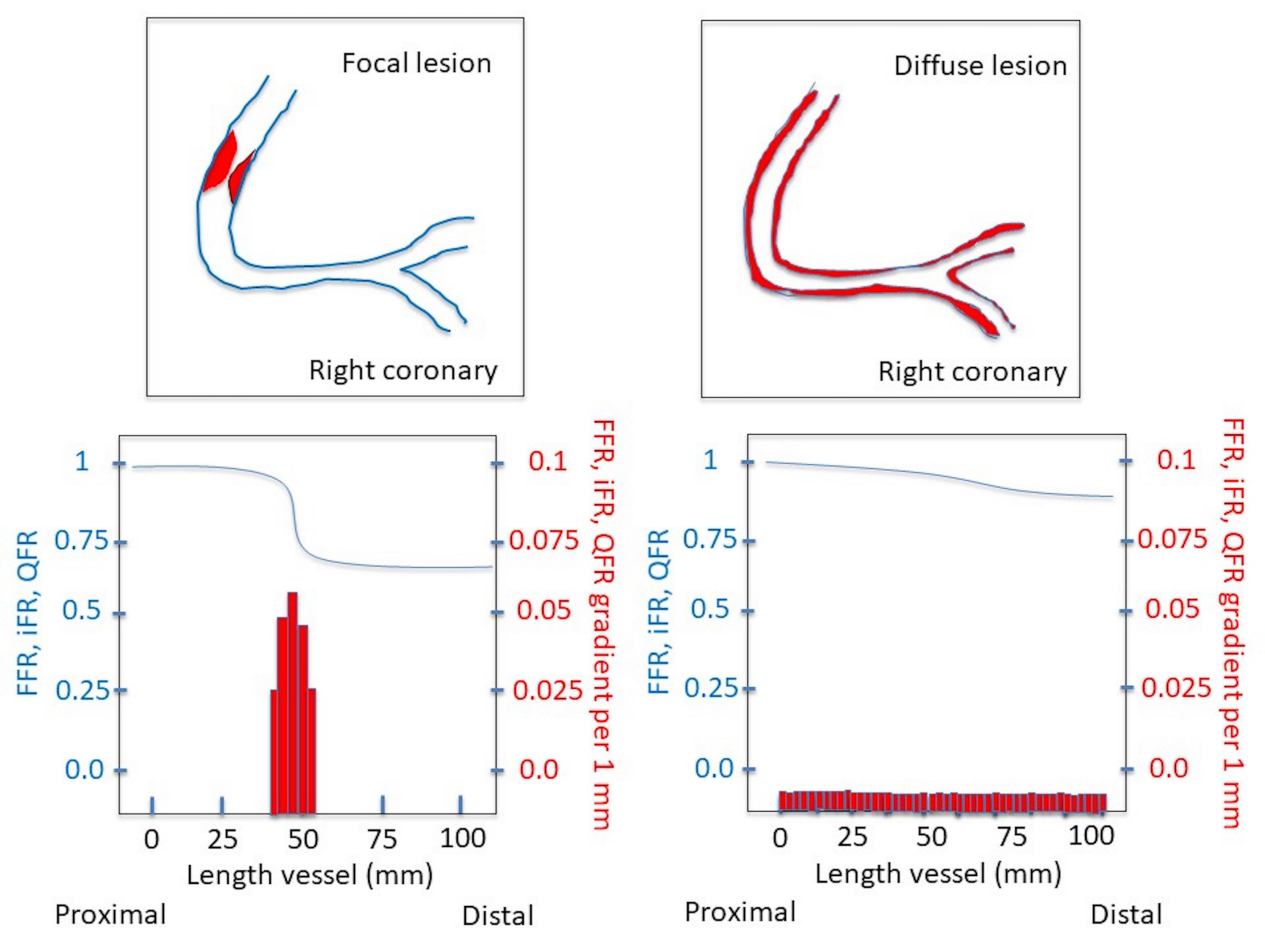
**Schematic illustration of the use of pullback index by FFR, iFR and QFR to characterize a coronary lesion**. FFR, fractional flow reserve; iFR, instantaneous wave-free ratio; QFR, Quantitative Flow Ratio.

MaxPPG is the maximum pressure gradient over 20 mm. Δ FFR vessel is the difference between the FFR values obtained along the complete length of the explored vessel (ostium to distal part). Functional disease length and total vessel length are derived from the vessel length explored by a motorized pullback system and FFR data on that vessel length. Functional disease length is determined as the length where the FFR drops >0.0015/mm. The system allows to obtain a real physiological map of the vessel. However, there are still limits such as the use of a motorized pullback system during prolonged adenosine infusions; index calculation is offline; and usefulness in clinical practice will require validation [85]. Feasibility of PPG by QFR was showed without guide pressure and motorized pullback system [86]. Finally, pullback technic with FFR, iFR, or QFR physiological map of the vessel very useful to program PCI and to evaluate its results [87, 88, 89] (Fig. 2).

However, the above mentioned pull back assessments are not yet supported by prospective randomized studies and cut-offs for the prediction of clinical events are not well defined. In this setting, the following sequence might reveal useful. A Pd/Pa <0.96 leads to FFR assessment, and if FFR <0.86, then FFR pullback and PPG are performed [90]. The use of virtual PCI is even more futuristic. Mapping of the artery before PCI and simulation of the PCI result is now possible. Virtual PCI is probably another step for precision medicine.

### 5.3 Identification of Patients at Risk

In the perspective of stratified medicine, coronary physiological indexes may represent theragnostic biomarkers, i.e., metrics that predict the therapeutic response. IMR measured after PCI allows for the identification of a group of patients with adverse prognosis when using a categorical value of 25 [91].

All indexes have diagnostic cutoffs and some also have prognostic cutoffs (Table 2). Coronary physiological assessment could help identify patients at risk for adverse events. The identification of patients at higher risk of adverse events will provide the possibility to implement specific therapies aimed at microvascular recovery and will lead to closer follow-up. However, randomized clinical trials are needed to validate these strategies.

**Table 2.** **Cut off and significations to coronary physiology assessment**.

	**Cut off**	**Meaning**
FFR	- Pre PCI ≤0.8	- Significant stenosis
	- Post PCI ≤0.86	- Worse prognosis
iFR	- Pre PCI ≤0.89	- Significant stenosis
	- Post PCI ≤0.89	- Worse prognosis
Other NHPR	- Pre PCI ≤0.89	- Significant stenosis
HSR	- Pre PCI <0.80 mmHg/cm/sec	- Significant stenosis
QFR, CAAS vFFR, FFRangio system	- Pre PCI ≤0.89	- Significant stenosis
	- Post PCI ≤0.89 for QFR	- Worse prognosis
FFR CT	- Pre PCI ≤0.8	- Significant stenosis
CFR	- Thermodilution <2	- Worse prognostic
	- Doppler <2.5	
IMR	- ≥25 mm Hg × seconds or units	- Microvascular dysfunction
	- Post PCI ≥25 mm Hg × seconds or units	- Worse prognostic
HMR	- ≥1.9 or ≥2.5 mmHg/cm/s	- Microvascular dysfunction
		- Predictor of recurrent chest pain
RRR	- No clear cut off <2.62 or <1.7 or <1.5	- CMVD diagnosis
		- Lower is worse for prognosis
Absolute CBF and resistance	- NA	- NA
MRR	- NA	- NA
IHDVPS, Pzf, WIA	- NA	- NA

Same abbreviations in Table 1.

## 6. Conclusions

The assessment of coronary physiology has become an indispensable technique for deciding on epicardial revascularization as well as for the exploration of the entire coronary tree and that of the coronary microcirculation in order to improve patient management. The range of available coronary physiological indexes has the potential to allow for individualized therapeutic strategies, therefore representing an additional step towards precision medicine.
